# Disability and physical activity in people with chronic disease receiving physiotherapy. A prospective cohort study

**DOI:** 10.3389/fspor.2022.1006422

**Published:** 2022-09-23

**Authors:** Lisa U. Tønning, Inger Mechlenburg, David H. Christiansen, Nils-Bo V. Andersen, Henriette H. Stabel, Asger R. Pedersen, Jørgen F. Nielsen, Bernd Grimm, Erhard Næss-Schmidt

**Affiliations:** ^1^Department of Orthopaedic Surgery, Aarhus University Hospital, Aarhus, Denmark; ^2^Department of Clinical Medicine, Aarhus University, Aarhus, Denmark; ^3^Department of Occupational Medicine, Danish Ramazzini Centre, Regional Hospital West Jutland, Herning, Denmark; ^4^Primary Health Care and Quality Improvement, Central Denmark Region, Viborg, Denmark; ^5^Hammel Neurorehabilitation Centre and University Research Clinic, Hammel, Denmark; ^6^Luxembourg Institute of Health, Human Motion, Orthopaedics, Sports Medicine, Digital Methods (HOSD) Platform, Strassen, Luxembourg

**Keywords:** chronic disease, physical activity, tri-axial accelerometry, modified Ranking Scale, physiotherapy

## Abstract

Chronic disease affects patients' disability and participation in activities of daily living. Longitudinal information on disability and physical activity is generally scarce in patients with chronic disease. The current study aimed to investigate if self-reported disability and physical activity changed in patients with chronic disease receiving physiotherapy. Furthermore, the aim was to assess if an improvement in self-reported disability was related to an increase in objectively measured physical activity and if an aggravation in self-reported disability was related to a decrease in physical activity. Seventy patients with either multiple sclerosis, Parkinson's disease, rheumatoid arthritis or stroke receiving free of charge physiotherapy were tested at baseline and 1 year later. Disability was measured with the self-reported modified Ranking Scale-9 Questionnaire and physical activity was objectively measured using tri-axial accelerometry. Neither self-reported disability nor physical activity changed among patients receiving 1 year of free of charge physiotherapy at group level. Furthermore, self-reported change of disability was not expressed with changes in objectively measured physical activity, indicating that the two measures represent two different constructs.

## Introduction

Chronic disease affects patients' disability and participation in activities of daily living. Moreover, chronic diseases constitute a major financial burden on both patients and society [1]. In Denmark, patients with chronic disease are entitled to free of charge physiotherapy (FCP), if they fulfill strict criteria [2]. Patients with multiple sclerosis (MS), Parkinson's disease (PD), rheumatoid arthritis (RA) and stroke experience functional disabilities which affect their daily living. Moreover, they constitute the four largest groups receiving FCP in Denmark [3], accounting for half of the total costs of FCP [2]. The purpose of FCP is to increase, maintain or to delay a decline in physical functioning [2].

Physical activity (PA) is a specific dimension of physical functioning [2]. PA has been defined as bodily movement produced by skeletal muscles that results in energy expenditure [4]. According to the World Health Organisation (WHO), PA is a complex behavior which should be described by measuring its four dimensions; frequency, intensity, time and type (F.I.T.T.) [5]. A systematic review regarding measurement of PA in patients with stroke found that accelerometry and behavioral mapping were the most commonly used methods to measure PA within this patient group [1]. Objective measure of PA with a validated algorithm may be the most appropriate method to measure and interpret the four dimensions of F.I.T.T. [6].

Longitudinal information on disability and PA is generally scarce in patients with chronic diseases [7] and it is unknown if self-reported change in disability is reflected in objectively measured PA. Self-reports are biased and may or may not reflect objective changes of clinical interest.

This longitudinal study aimed to investigate if self-reported disability and PA in patients with chronic disease receiving FCP, were changed at 1 year follow up. Furthermore, we aimed to assess if an improvement in self-reported disability was related to an increase in objectively measured PA and if an aggravation in self-reported disability was related to a decrease in PA.

## Materials and methods

### Participants

Patients were invited to participate in this prospective study, through another study conducting a survey in 10 out-patient physiotherapy clinics in the Central Denmark Region [8]. The data collection for the present study took place in the autumn 2018 and the autumn 2019. The inclusion criteria were: age ≥18 years, diagnosed with MD, PD, RA or stroke and entitled to receive FCP. Patients were excluded if they were: unable to get up from a chair by themselves or unable to fully understand written questionnaires. A group of trained assessors assessed all patients at baseline and 1 year later, at the out-patient physiotherapy clinics. All patients gave written and oral consent to participate. According to The Central Denmark Region Committee on Health Research Ethics, ethical approval of this study was not needed (request: 56/2018). The Danish Data Protection Agency (j. nr. 1-16-02-757-17) approved the study.

### Free of charge physiotherapy

In Denmark patients with a chronic disease are entitled to FCP according to strict criteria administered by the general practitioner or a medical specialist. The criteria for receiving FCP include having (1) one of the 43 diagnoses defined in the FCP program, (2) a severe physical disability or progressive disease, and (3) a prognosis which is likely to last for at least 5 years [2]. In addition, patients with less severe disability but an abnormal function of the sensory-motor or nervous system are entitled to receive FCP [2]. The content of FCP is broad and depends on the individual patient's need. It often includes group-based training, individual sessions and home-based training.

### Self-reported disability

The modified Ranking Scale-9 Questionnaire (mRS-9Q) measures functional outcome and categorizes level of disability or independence [9]. Following completion of the mRS-9Q, the patient is categorized into one of the seven possible disability categories (ranging from no symptoms to death). The mRS-9Q has been found to be a reliable and responsive tool for measurement of disability [9]. The mRS-9Q was in relation to our study translated and culturally adapted into Danish according to international guidelines; validation studies are ongoing. In the current study, the Danish version of the mRS-9Q was used to assess self-reported disability at baseline and at 12-months follow-up. Differences in scores of mRS-9Q between baseline and follow-up were used to categorize the patients into three groups; improved disability (baseline score > follow-up score), unchanged disability (baseline score = follow-up score) or aggravated disability (baseline score < follow-up score).

### Objective measurement of physical activity

PA during day and night was measured continuously with tri-axial accelerometers (AX3, Axivity Ltd., Newcastle, UK). The accelerometer measures accelerations in three dimensions at 100 Hz. The accelerometer was placed on the patients' right thigh between the major trochanter and the lateral femoral condyle [10]. Patients were asked to wear the accelerometer for at least seven consecutive days [11].

### Data analysis

Data from the accelerometer were downloaded using OMGUI Configuration and Analysis Tool (Version 1.0.0.43, Newcastle, UK) and divided into days using a MatLab (MatLab R2019b, MathWorks, Natick, USA) script designed for this purpose. After separation of data into days, each day was analyzed using a validated algorithm described by Lipperts et al. [12] and validated for impaired slow walking patients [13]. The algorithm calculated the number of steps, number of transfers from sitting to standing, number of stair climbing events and the percentage of time spent standing, walking or sedentary. For every bout of walking, the intensity was quantified by calculating the cadence of the stepping activity (steps/min) as a proxy of walking speed. Patients with missing accelerometer data at either baseline or follow-up where excluded. Patients with data available for <3 days were excluded [10, 14].

### Statistics

No formal sample size calculation was conducted for this study. Continuous data were assessed for normality using histograms and probability plots. Parametric outcome was reported as means with standard deviations (SD), while non-parametric outcomes were reported as median with interquartile range (IQR). All categorical outcomes were presented as number of events with percentages of total events. Baseline characteristics between included and excluded patients was performed using the Student *T*-test. PA parameters were divided by the number of days wearing the accelerometer. Since all PA parameters and mRS-9Q displayed a non-normal distribution, Wilcoxon signed-rank test was used to investigate a possible change in the dimensions of F.I.T.T., as well as in the mRS-9Q, from baseline to follow-up. In addition, data were logarithmic transformed to achieve normality and the association between a change in disability and a change in PA was analyzed using linear regression analysis, reported as the *F*-value (F), *P*-value (P), and the Coefficient of Determination (*R*^2^). Multiple linear regression analysis was further applied to check if adjusting for body mass index (BMI), gender, age and use of walking aids would change the results. Statistical analysis was performed using STATA 16.1 (StataCorp, College Station, TX, USA).

## Results

**Figure 1:**
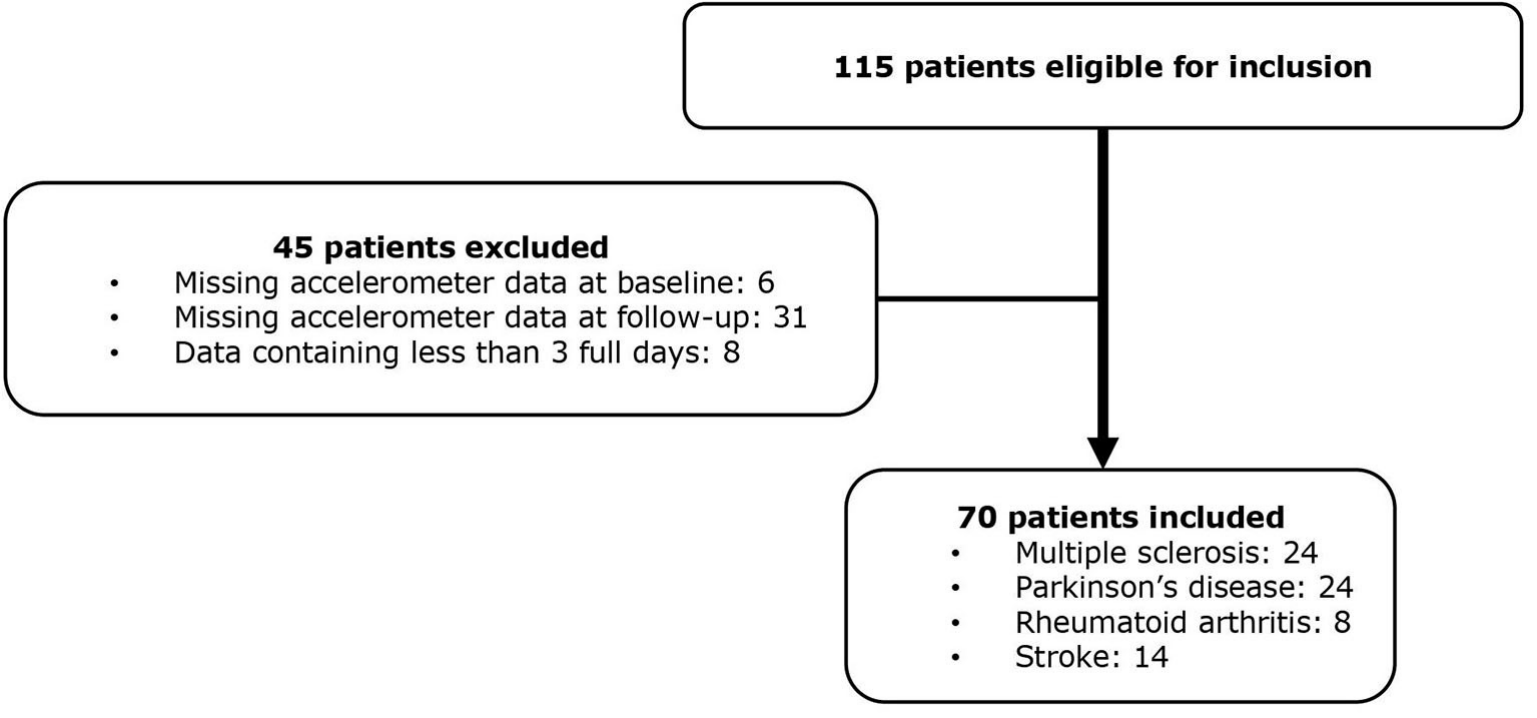
Flow chart of included patients.

A total of 115 patients receiving FCP were included at baseline, 45 patients were excluded, leaving 70 patients with complete data for analysis (Figure 1). The dropout analysis showed no statistically significant difference regarding baseline characteristics (Supplementary Table 1). The average days of accelerometer recording was 6.4 at baseline (SD 1.6) and 6.7 at follow-up (SD 1.4). Baseline characteristics of the entire cohort and each chronic disease are presented in Table 1. Baseline characteristics for the three disability groups are found in the Supplementary Table 2. Patients in the MS group (56 ± 8.9, mean ± SD) were on average 10 years younger than the patients in the PD (67 ± 7.8) and stroke group (65 ± 12.7), and were more likely to use assistive walking devices (42%).

**Table 1:** Baseline characteristics.

	**All patients (*n* = 70)**	**Multiple sclerosis** ** (*n* = 24)**	**Parkinson's disease** ** (*n* = 24)**	**Rheumatoid arthritis** ** (*n* = 8)**	**Stroke** ** (*n* = 14)**
Age (years), mean (SD)	61.4 (11.4)	55.5 (8.9)	66.5 (7.8)	57.0 (16.1)	65.1 (12.7)
Gender, number of male (%)	28 (40.0)	5 (20.8)	15 (62.5)	1 (12.5)	7 (50.0)
BMI, mean (SD)	27.1 (5.0)	27.2 (5.0)	25.8 (4.4)	29.2 (6.7)	27.9 (4.8)
Years since diagnosis, median (IQR)	9 (5; 13)^a^	10.5 (6; 18)^b^	7 (4; 9)^c^	11.5 (8; 16.5)	8 (2; 13)^d^
Living alone, *n* (%)	10 (14.3)	2 (8.3)	3 (12.5)	2 (25.0)	3 (21.4)
Use of assistive walking device, *n* (%)	16 (22.9)	10 (41.7)	0 (0)	2 (25.0)	4 (28.6)
**Education**, ***n*** **(%)**
Primary school	6 (8.8)	3 (12.5)	3 (13.6)	0 (0)	0 (0)
High school or vocational education (< 2.5 years)	34 (50.0)	14 (58.3)	9 (40.9)	3 (37.5)	8 (57.1)
2.5–4 years higher education	14 (20.6)	2 (8.3)	5 (22.7)	4 (50.0)	3 (21.4)
>4 years higher education	10 (14.7)	2 (8.3)	4 (18.2)	1 (12.5)	3 (21.4)
Other	4 (5.9)	3 (12.5)	1 (4.6)	0 (0)	0 (0)
**Employment status**, ***n*** **(%)**
Working	9 (12.9)	3 (12.5)	5 (20.8)	1 (12.5)	0 (0)
Flex job	9 (12.9)	6 (25.0)	2 (8.3)	1 (12.5)	0 (0)
On pension	46 (65.7)	13 (54.2)	17 (70.8)	3 (37.5)	13 (92.9)
Other	6 (8.6)	2 (8.3)	0 (0)	3 (37.5)	1 (7.1)

^a^59 patients.

^b^22 patients.

^c^18 patients.

^d^11 patients.

SD, standard deviation; BMI, body mass index; IQR, interquartile range.

The distribution of change in disability is presented in Table 2. All patients with RA reported a better or same mRS-9Q score at follow-up compared to baseline. Only five patients changed more than one point on the mRS-9Q and all five changed only two points. Three patients improved; one changed from a score of two to zero, one changed from three to one and one changed from a score of four to two. Two patients got worse; one changed score from zero to two and one changed from one to three. There was no statistically significant difference between mRS-9Q measured at baseline and follow-up (*p* = 0.96).

**Table 2:** The modified Ranking Scale-9 Questionnaire measured at baseline and at 1 year follow-up in patients with four chronic diseases.

	**All patients** **(*****n*** = **70)**		**Multiple sclerosis** **(*****n*** = **24)**		**Parkinson's disease** **(*****n*** = **24)**		**Rheumatoid arthritis** **(*****n*** = **8)**		**Stroke** **(*****n*** = **14)**	
**Definition, *n* (%)**	**Baseline**	**Follow-up**	**Baseline**	**Follow-up**	**Baseline**	**Follow-up**	**Baseline**	**Follow-up**	**Baseline**	**Follow-up**
No symptoms	3 (4.3)	6 (8.6)	2 (8.3)	3 (12.5)	1 (4.2)	2 (8.3)	0 (0)	0 (0)	0 (0)	0 (0)
No significant disability	10 (14.3)	7 (10.0)	3 (12.5)	1 (4.2)	5 (20.8)	6 (25.0)	0 (0)	0 (0)	2 (14.3)	1 (4.1)
Slight disability	31 (44.3)	28 (40.0)	7 (29.2)	6 (25.0)	11 (45.8)	9 (37.5)	3 (37.5)	4 (50.0)	10 (71.4)	9 (64.3)
Moderate disability	20 (28.6)	24 (34.3)	11 (45.8)	12 (50.0)	4 (16.7)	5 (20.8)	3 (37.5)	3 (37.5)	2 (14.3)	4 (28.6)
Moderately severe disability	6 (8.6)	5 (7.1)	1 (4.2)	2 (8.3)	3 (12.5)	2 (83)	2 (25.0)	1 (12.5)	0 (0)	0 (0)
Severe disability	0 (0)	0 (0)	0 (0)	0 (0)	0 (0)	0 (0)	0 (0)	0 (0)	0 (0)	0 (0)
Dead	0 (0)	0 (0)	0 (0)	0 (0)	0 (0)	0 (0)	0 (0)	0 (0)	0 (0)	0 (0)

Table 3 gives an overview of the PA parameters from baseline to follow-up for the entire cohort and for each chronic disease. In general, PA did not change from baseline to follow-up. This applied to all four disease groups. Categorizing patients based on their change in self-reported disability of improvement, no change or aggravation, did not illustrate any association to change in PA in regard to increase or decrease of PA (Table 4). Change of disability did not predict PA in terms of steps [*F*_(2, 67)_ = 1.07, *P* = 0.35, *R*^2^ = 0.03], average cadence [*F*_(2, 67)_ = 0.71, *P* = 0.49, *R*^2^ = 0.02], inclined walking steps [*F*_(2, 67)_ = 0.71, *P* = 0.46, *R*^2^ = 0.02] and sit-to-stand-transfers [*F*_(2, 67)_ = 0.30, *P* = 0.74, *R*^2^ = 0.01] from baseline to follow-up. Adjusting for BMI, gender, age and use of walking aids did not change the results.

**Table 3:** Physical activity per day of patients with the four chronic diseases described by the dimensions of frequency, intensity, time, and type.

**Dimension**	**Parameter, per day**	**Baseline,** ** median (IQR)**	**Follow-up,** ** median (IQR)**	**Change^a^,** ** median (IQR)**	***P*-value**
**All patients (*****n*** **=** **70)**
Frequency (number)	Steps	5,348 (3,844; 7,894)	4,977 (3,817; 7,260)	−234 (−1,140; 769)	0.33
	Inclined walking steps	254 (104; 516)	217 (85; 423)	−5 (−137; 91)	0.49
Intensity (steps/min)	Average cadence	89.6 (83.7; 95.2)	89.6 (85.8; 97.5)	0.8 (−3.1; 3.4)	0.24
Time (hours)	Wear time	15.5 (15.0; 16.1)	15.4 (14.8; 16.0)	−0.3 (−0.8; 0.6)	0.17
	Walking	1.4 (1.0; 1.9)	1.3 (1.0; 1.8)	−0.1 (−0.3; 0.2)	0.15
	Standing	3.7 (2.8; 4.9)	3.8 (2.8; 5.2)	0.1 (−0.4; 0.8)	0.18
	Sendentary	10.0 (8.3; 11.2)	9.7 (8.3; 11.3)	−0.3 (−1.0; 0.7)	0.22
Type (number)	Sit to stand transfers	46 (39; 54)	46 (37; 58)	−1 (−5; 5)	0.59
**Multiple sclerosis (*****n*** **=** **24)**
Frequency (number)	Steps	4,839 (3,269; 8,061)	5,134 (3,672; 7,212)	−3 (−1,082; 895)	0.95
	Inclined walking steps	210 (100; 545)	173 (72; 338)	−16 (−110; 77)	0.53
Intensity (steps/min)	Average cadence	87.1 (81.6; 95.6)	88.1 (80.8; 98.3)	1.0 (−2.0; 2.8)	0.42
Time (hours)	Wear time	15.4 (15.0; 15.9)	15.2 (14.5; 15.9)	−0.4 (−0.8; 0.5)	0.35
	Walking	1.3 (0.9; 1.9)	1.3 (1.0; 1.8)	−0.03 (−0.2; 0.2)	0.69
	Standing	3.8 (3.0; 4.3)	3.3 (2.6; 4.4)	−0.3 (−0.5; 0.5)	0.53
	Sendentary	10.0 (9.1; 11.1)	10.0 (9.1; 11.7)	0.0004 (−0.8; 0.9)	0.96
Type (number)	Sit to stand transfers	50 (43; 62)	48 (42; 61)	−2 (−5; 4)	0.59
**Parkinson's disease (*****n*** **=** **24)**
Frequency (number)	Steps	6,591 (4,575; 8,773)	5,412 (4,008; 8,865)	−533 (−1,806; 699)	0.13
	Inclined walking steps	315 (181; 536)	348 (217; 736)	24 (−159; 397)	0.35
Intensity (steps/min)	Average cadence	94.4 (89.6; 98.8)	95.6 (88.4; 101.6)	1.9 (−1.9; 8.2)	0.15
Time (hours)	Wear time	15.7 (15.3; 16.4)	15.7 (15.2; 16.2)	−0.2 (−0.7; 0.5)	0.41
	Walking	1.5 (1.1; 2.0)	1.3 (0.9; 2.2)	−0.1 (−0.5; 0.1)	0.09
	Standing	4.6 (3.4; 5.5)	4.9 (3.4; 5.4)	0.1 (−0.3; 1.1)	0.38
	Sendentary	8.6 (8.0; 10.8)	9.1 (8.1; 10.4)	−0.2 (−0.9; 0.5)	0.51
Type (number)	Sit to stand transfers	45 (38; 53)	42 (36; 57)	−2 (−7; 4)	0.48
**Reumatoid arthritis (*****n*** **=** **8)**
Frequency (number)	Steps	4,739 (2,954; 5 876)	4,677 (3 809; 7,011)	855 (−3; 1,941)	0.16
	Inclined walking steps	301 (146; 467)	102 (81; 262)	−122 (−288; 14)	0.07
Intensity (steps/min)	Average cadence	87.7 (84.7; 90.3)	89.4 (87.2; 90.2)	2.2 (−5.8; 4.8)	0.89
Time (hours)	Wear time	15.1 (14.6; 16.2)	15.5 (14.7; 16.3)	0.3 (−0.4; 0.9)	0.67
	Walking	1.3 (0.7; 1.6)	1.2 (1.0; 1.6)	0.2 (−0.03; 0.3)	0.26
	Standing	3.7 (2.5; 4.9)	3.4 (2.7; 5.9)	0.4 (−0.01; 1.0)	0.21
	Sendentary	10.0 (7.8; 12.4)	10.8 (8.2; 11.4)	−0.9 (−1.1; 0.2)	0.21
Type (number)	Sit to stand transfers	43 (39; 50)	47 (34; 60)	0.3 (−3; 9)	1.00
**Stroke (*****n*** **=** **14)**
Frequency (number)	Steps	4,724 (4,155; 6,956)	4,760 (4,260; 6,202)	−247 (−1,302; 338)	0.30
	Inclined walking steps	90 (31; 279)	101 (29; 195)	−5 (−97; 12)	0.36
Intensity (steps/min)	Average cadence	87.0 (80.3; 91.2)	88.1 (76.6; 89.8)	−0.4 (−3.9; 0.3)	0.22
Time (hours)	Wear time	15.0 (15.0; 15.7)	15.0 (13.8; 15.5)	−0.5 (−1.3;−0.03)	0.27
	Walking	1.4 (1.0; 1.7)	1.3 (1.0; 1.6)	−0.1 (−0.3; 0.1)	0.20
	Standing	3.5 (2.2; 4.7)	3.9 (2.1; 4.4)	0.4 (−0.3; 1.2)	0.10
	Sendentary	10.7 (8.7; 11.4)	9.8 (8.4; 11.5)	−0.7 (−1.3; 0.7)	0.30
Type (number)	Sit to stand transfers	39 (32; 46)	41 (33; 48)	1 (−5; 6)	0.47

^a^Improvement is calculated as follow-up score minus baseline score. IQR, interquartile range.

**Table 4:** Physical activity per day in relation to the change in disability described by the dimensions of frequency, intensity, time, and type.

**Dimension**	**Parameter**	**Baseline,** ** median (IQR)**	**Follow-up,** ** median (IQR)**	**Improvement^a^,** ** median (IQR)**	***P*-value**
**Improved disability (*****n** **=*** **14)**
Frequency (number)	Steps	6,067 (3,432; 8,904)	5,081 (4,177; 8,017)	−220 (−1,316; 797)	0.40
	Inclined walking steps	258 (97; 516)	201 (105; 366)	−24 (−134; 47)	0.55
Intensity (steps/min)	Average cadence	91.5 (85.3; 96.8)	89.7 (86.9; 98.1)	1.6 (−3.5; 2.4)	0.73
Time (hours)	Wear time	15.7 (15.2; 16.4)	16.2 (15.6; 16.7)	0.1 (−0.4; 0.8)	0.64
	Walking	1.5 (0.8; 2.1)	1.3 (0.9; 1.8)	0.1 (−0.3; 0.1)	0.88
	Standing	3.3 (2.3; 5.1)	3.9 (2.5; 5.4)	0.3 (−0.2; 1.0)	0.07
	Sendentary	10.2 (8.4; 12.5)	10.6 (8.5; 11.8)	−0.1 (−0.9; 0.3)	0.68
Type (number)	Sit to stand transfers	48 (40; 58)	51 (40; 66)	−0.4 (−3; 6)	0.93
**Unchanged disability (*****n** **=*** **42)**
Frequency (number)	Steps	5,151 (3,878; 8,109)	5,031 (4,260; 7,260)	40 (−1,046; 769)	0.92
	Inclined walking steps	231 (104; 505)	242 (85; 541)	3 (−137; 127)	1.00
Intensity (steps/min)	Average cadence	89.7 (82.9; 95.3)	89.5 (86.6; 95.0)	0.4 (−3.9; 3.8)	0.63
Time (hours)	Wear time	15.5 (15.0; 16.1)	15.2 (14.8; 15.9)	−0.4 (−0.8; 0.5)	0.10
	Walking	1.4 (1.0; 1.9)	1.3 (1.0; 1.7)	−0.1 (−0.2; 0.2)	0.53
	Standing	3.9 (3.1; 4.9)	3.8 (2.8; 5.1)	0.02 (−0.4; 0.5)	0.66
	Sendentary	10.0 (8.3; 11.2)	9.7 (8.4; 10.9)	−0.5 (−1.2; 0.8)	0.26
Type (number)	Sit to stand transfers	44 (38; 53)	45 (37; 57)	−1 (−4; 4)	0.91
**Aggravated disability (*****n** **=*** **14)**
Frequency (number)	Steps	5,155 (2,747; 7,365)	4,291 (3,318; 7,083)	−705 (−1,267; 759)	0.27
	Inclined walking steps	256 (64; 689)	187 (34; 328)	−22 (−330; 66)	0.25
Intensity (steps/min)	Average cadence	84.7 (81.7; 94.5)	89.3 (81.9; 98.8)	2.1 (−0.6; 3.4)	0.07
Time (hours)	Wear time	15.2 (15.0; 15.6)	15.3 (14.5; 15.8)	−0.3 (−1.0; 0.3)	0.47
	Walking	1.3 (0.9; 1.6)	1.0 (0.8; 1.8)	−0.2 (−0.4; 0.1)	0.14
	Standing	3.7 (3.4; 4.8)	3.9 (3.1; 5.5)	0.07 (−0.4; 1.0)	0.55
	Sendentary	9.4 (8.6; 10.99	9.1 (8.3; 11.6)	−0.05 (−0.4; 0.4)	0.83
Type (number)	Sit to stand transfers	45 (41; 56)	47 (37; 55)	−3 (−10; 5)	0.22

^a^Improvement is calculated as follow-up score minus baseline score. IQR, interquartile range.

## Discussion

Overall there were no statistically significant changes in self-reported disability or any PA parameters at group level, even though 40% of the patients had changed their self-reported level of disability. Change in self-reported disability quantified by the mRS-9Q was not related to objectively measured PA parameters in this cohort of patients with chronic diseases.

Of the eligible patients with complete data, 61% participated in the 1 year follow-up. On average, the accelerometers were worn for almost a week, meaning that the results included both weekdays and weekend, hence reflecting possible variations in PA and activity behavior which strengthen the results of the study.

There were no statistically significant differences in the mRS-9Q score from baseline to follow-up within this cohort. This could be due to changes going in both directions as well as a result of the FCP, where a realistic aim is for patients to maintain their function. Another reason could be that the categories of mRS-9Q are very rough and small changes in disability are thus not detected.

A systematic review from 2011 by Tudor-Locke et al. estimated that the minimum amount of daily steps is 7,000–8,000 for healthy adults [15]. The majority of patients in our cohort thus had a lower number of daily steps than what is recommended for maintaining general health in adults. In addition, the median average cadence for the total cohort was also lower than the 100 steps/min, which was described by Tudor-Locke et al. as being a reasonable floor value of moderate intensity walking in healthy participants [15]. As such, it makes sense that our patients were found eligible for receiving FCP due to the disability caused by their disease. Kujala et al. also found that chronic diseases (including RA, PD and stroke) were associated with a lower level of PA [16]. A systematic review from 2017 by Fini et al. found that the combined average number of steps more than 6 months after a stroke event was 4,078 steps per day measured in 1,280 patients from 32 different studies [7]. The 14 patients with stroke in our cohort had a median number of daily steps which were higher than in the study by Fini et al. [7]. Whether FCP contributed to the higher number of daily steps is difficult to conclude based on this small cohort. Opposite, the median years since diagnosis in our cohort was 9 years which is relatively longer than in the study population by Fini et al. [7].

### Limitations

This study has certain limitations. First, as described earlier the aim of FCP is to increase, maintain or delay a decline in function and PA, however since we did not include a control group, we do not know how the PA level of these patients would have been if they had not received FCP during a 12-month period. Consequently, we cannot determine if the treatment has been successful or not. Also, we do not know the exact content of interventions offered during FCP, the specific purpose for each patient to attend FCP or how many times each patient received FCP. However, the patients in our study were assigned to the FCP programme assuming that their PA level would decline or they would be more disabled over time. Since 80% of the patients reported an unchanged or improved level of disability, the FCP seems to have contributed with preserved PA for these patients.

Second, the individual changes in the objective measured PA did not correspond with self-reported changes in disability measured with the mRS-9Q, as self-reported disability may not be a precise method to express PA and the two measurements might measure different constructs [17, 18]. In addition, the four diseases are all progressive diseases and we do not know whether this influences the self-reported disability and the PA parameters similarly. A patient may have experienced a decline in disability and thus needs to use an assistive walking device but is otherwise able to perform the same activities as earlier. This would change the mRS-9Q score from not using an assistive device to next level of using assistive device; this, however, does not necessarily result in a similar change in PA parameters.

Third, we did not measure complete PA as we did not categorize activities as housework, garden work, etc. Nevertheless, we used a validated algorithm to collect information on the different F.I.T.T dimensions in relation to PA within four disease groups and three disability groups. The number of daily steps does not necessarily describe the patients' actual capacity for performing PA or disability level completely, as this information may be influenced by practical issues, such as the size of house and usage of an assistive walking device. By collecting information on the different F.I.T.T dimensions, we have been able to expand the description of the different components of PA instead of e.g., only a single number of daily steps. However, we recognize that we have not been able to collect all the F.I.T.T dimensions, such as the distribution of walking bouts or the sit-to-stand transfer time and intensity.

Fourth, the timeframe of the follow-up is a limitation of this study. Since the four diseases in this study are all progressive, the aim of the FCP could change several times during a year. In addition, patients with progressive diseases may decline in function despite receiving physiotherapy. Variations caused by different disease progression and training intensity over the year may explain the null finding at group level. When designing this study, it was decided to collect data at the same time of the year to avoid confounding of seasonal changes in PA behavior and to obtain long term follow-up data.

Fifth, we used the Danish version of the mRS-9Q which has recently been validated but the final results from this work have not yet been published. Nevertheless, the English version of the questionnaire has been found reliable and responsive among patients with stroke [9] and the choice of a 1-point change is stated by Dromerick et al. to be reasonable since the categories are quite broad [19]. Fourth, the number of included patients is rather low to allow a separate sub-group analysis, especially for patients with RA and stroke. This might have caused a type 2 error. The small number of patients limits the generalizability of the study for specific diseases. Fifth, there is presumably some selection bias in the study. Even though the drop-out analysis showed no difference in baseline characteristics some of the severely disabled patients may have declined to participate due to lack of resources following disease progression within the 1 year follow-up time.

## Conclusion

This longitudinal study found no overall changes in self-reported disability or PA in a cohort of patients with MS, PD, rheumatoid arthritis and stroke receiving FCP for 1 year. Moreover, self-reported change in disability was not related to change in accelerometer assessed PA among the patients.

## Data availability statement

The raw data supporting the conclusions of this article will be made available by the authors, without undue reservation.

## Ethics statement

The studies involving human participants were reviewed and approved by the Central Denmark Region Committee on Health Research Ethics. The patients/participants provided their written informed consent to participate in this study.

## Author contributions

LT, IM, DC, N-BA, AP, JN, BG, and EN-S have contributed substantially to the conception or the design of the manuscript. LT, IM, HS, AP, BG, and EN-S performed the analyses and interpreted the data. LT, IM, and EN-S have revised the manuscript critically. All authors have participated in the drafting of the manuscript and read and approved the final version of the manuscript.

## Funding

This study was supported by Fysioterapipraksisfonden and Søster and Verner Lipperts Foundation.

## Conflict of interest

The authors declare that the research was conducted in the absence of any commercial or financial relationships that could be construed as a potential conflict of interest.

## Publisher's note

All claims expressed in this article are solely those of the authors and do not necessarily represent those of their affiliated organizations, or those of the publisher, the editors and the reviewers. Any product that may be evaluated in this article, or claim that may be made by its manufacturer, is not guaranteed or endorsed by the publisher.

## Abbreviations

FCP: Free of Charge Physiotherapy
F.I.T.T., Frequency, Intensity: Time and Type
IQR: Interquartile Range
mRS-9Q: the modified Ranking Scale-9 Questionnaire
MS: Multiple sclerosis
PA: Physical Activity
PD: Parkinson's disease
RA: Rheumatoid Arthritis
SD: Standard Deviations (SD)
WHO: the World Health Organisation.
