# Supplementary material for: Disability and physical activity in people with chronic disease receiving physiotherapy. A prospective cohort study
Source: Front Sports Act Living. 2022 Sep 23;4:1006422. doi: 10.3389/fspor.2022.1006422 (PMC9537493; doi:10.3389/fspor.2022.1006422)
Supplement: Supplementary file 1 [file Data_Sheet_1.docx]

**SUPPLEMENTARY TABLES**

Supplementary information for review and online publication only.

Supplementary Table 1. Drop out analysis.

|  | | | | | | |
| --- | --- | --- | --- | --- | --- | --- |
|  | Baseline | | | Follow-up | | |
|  | Included patients (n=70) | Excluded patients (n=45) | P-value | Included patients (n=70) | Excluded patients (n=15) | P-value |
| Age (years), mean (SD) | 61.4 (11.4) | 62.7 (13.4) | 0.37 | 62.4 (11.5) | 61.5 (12.2) | 0.69 |
| Gender, number of male (%)^a^ | 28 (40.0) | 20 (45.5) | 0.57 |  |  |  |
| BMI, mean (SD) | 27.1 (5.0) | 27.7 (5.6) | 0.80 | 26.5 (4.7)^b^ | 27.6 (6.1) | 0.67 |
| Years since diagnosis, median (IQR) | 9 (5;13)^c^ | 11 (6;18)^d^ | 0.39 | 9 (5;13)^c^ | 12 (5;22) | 0.17 |
| Living alone, n (%) | 10 (14.3) | 8 (18.2) | 0.58 | 12 (17.4)^b^ | 3 (21.4)^e^ | 0.72 |
| Use of assistive walking device, n (%) | 16 (22.9) | 17 (37.8) | 0.08 | 23 (32.9) | 4 (26.7) | 0.64 |
| Education, n (%)^a^ |  |  | 0.26 |  |  |  |
| None | 6 (8.8) | 6 (14.0) |  |  |  |  |
| Short <2.5 years | 34 (50.0) | 22 (51.2) |  |  |  |  |
| Medium 2.5-4 years | 14 (20.6) | 10 (23.3) |  |  |  |  |
| Long >4 years | 10 (14.7) | 1 (2.3) |  |  |  |  |
| Other | 4 (5.9) | 4 (9.3) |  |  |  |  |
| Employment status, n (%)^a^ |  |  | 0.38 |  |  |  |
| Working | 9 (12.9) | 2 (12.9) |  |  |  |  |
| Flex job | 9 (12.9) | 7 (15.9) |  |  |  |  |
| On pension | 46 (65.7) | 33 (75.0) |  |  |  |  |
| Other | 6 (8.6) | 8 (7.0) |  |  |  |  |
| ^a^follow-up information missing. ^b^69 patients. ^c^59 patients. ^d^43 patients. ^e^14 patients. SD = standard deviation. BMI = body mass index. IQR = interquartile range. | | | | | | |

Supplementary Table 2. Baseline characteristics for each of the disability groups.

|  | | | |
| --- | --- | --- | --- |
|  | Improved disability (n=14) | Unchanged disability (n=42) | Aggravated disability (n=14) |
| Age (years), mean (SD) | 63.4 (11.6) | 61.3 (10.5) | 59.6 (14.1) |
| Gender, number of male (%) | 6 (42.9) | 17 (40.5) | 5 (35.7) |
| BMI, mean (SD) | 26.3 (5.4) | 27.6 (5.1) | 26.4 (4.4) |
| Years since diagnosis, median (IQR) | 7 (6;13)^e^ | 9.5 (8;13)^f^ | 5 (2;10.5)^g^ |
| Living alone, n (%) | 3 (21.4) | 6 (14.3) | 1 (7.1) |
| Use of assistive walking device, n (%) | 2 (14.3) | 12 (28.6) | 2 (14.3) |
| Education, n (%) | |  |  |
| None | 1 (7.1) | 4 (9.8) | 1 (7.7) |
| Short <2.5 years | 7 (50.0) | 20 (48.8) | 7 (53.9) |
| Medium 2.5-4 years | 2 (14.3) | 9 (22.0) | 3 (23.1) |
| Long >4 years | 3 (21.4) | 6 (14.6) | 1 (7.7) |
| Other | 1 (7.1) | 2 (4.9) | 1 (7.7) |
| Employment status, n (%) | | |  |
| Working | 3 (21.4) | 6 (14.3) | 0 (0) |
| Flex job | 2 (14.3) | 5 (11.9) | 2 (14.3) |
| On pension | 9 (64.3) | 27 (64.3) | 10 (71.4) |
| Other | 0 (0) | 4 (9.5) | 2 (14.3) |
| The disability groups are categorised according to the change in mRS-9Q from baseline to follow-up. ^a^59 patients. ^b^22 patients. ^c^18 patients. ^d^11 patients. ^e^13 patients. ^f^34 patients. ^g^12 patients. SD = standard deviation. BMI = body mass index. IQR = interquartile range. | | | |
